# Clinical Characteristics and Follow-Up of Seizures in Children With Anti-NMDAR Encephalitis

**DOI:** 10.3389/fneur.2021.801289

**Published:** 2022-01-05

**Authors:** Jianzhao Zhang, Jing Sun, Ping Zheng, Shuo Feng, Xiaoli Yi, Haitao Ren, Qian Chen

**Affiliations:** ^1^Department of Pediatric Neurology, Children's Hospital Affiliated to the Capital Institute of Pediatrics, Beijing, China; ^2^Department of Neurology, Peking Union Medical College Hospital, Chinese Academy of Medical Science, Beijing, China

**Keywords:** encephalitis, epilepsy, children, NMDA–receptor, seizure

## Abstract

**Objective:** To analyze the seizure characteristics of children with anti-NMDAR encephalitis.

**Methods:** This was a retrospective analysis of 50 children with anti-NMDAR encephalitis between July 1, 2013, and July 1, 2019.

**Results:** Fifty children with anti-NMDAR encephalitis were included in this study, of which 34 (68.0%) had seizures. During the follow-up, three patients with anti-NMDAR encephalitis secondary to herpes simplex virus encephalitis had persistent seizures. The average duration of seizures in the remaining patients was 14.6 days (range 1–47 days). Compared to patients without seizures, those with seizures were more likely to experience consciousness disturbances (*p* = 0.008) and epileptic form discharge on electroencephalograms (*p* = 0.002). The Glasgow coma scale scores (*p* = 0.014), and Rankin scale scores (*p* = 0.019) were also different. The cranial MRI findings of children were reviewed, and clinical characteristics were compared between children without cranial lesions and those with lesions in the limbic system and neocortex. Compared to children in the non-lesion group, children with lesions in the limbic system and neocortex had a higher incidence of status epilepticus. Further, children in the limbic system and neocortical lesions groups were more likely to be taking anti-seizure medications (ASMs) and receive second-line drugs.

**Conclusion:** Long-term oral ASMs are not recommended for most children with anti-NMDAR encephalitis. Children with involvement of the limbic system and neocortex are prone to status epilepticus, and sequelae of epilepsy may remain when the neocortex is involved.

## Introduction

Since the discovery of the anti-NMDAR antibody in 2007 (1), reports of anti-NMDAR encephalitis have gradually increased, and it is currently the most common cause of autoimmune encephalitis (2, 3). Seizures are often the presenting symptom in children with autoimmune encephalitis, occurring in up to 72% of cases (4). Status epilepticus and cluster seizures are also common. However, data regarding the seizure characteristics of children with anti-NMDAR encephalitis, as well as anti-epileptic treatments and long-term follow-up results, are limited. Further, there are no universal guidelines for the management of seizures in children with anti-NMDAR encephalitis. This study retrospectively analyzed the clinical features and efficacy of anti-seizure medication (ASM) in children with anti-NMDAR encephalitis and summarizes our experience to provide a basis for the personalized treatment of seizures in children with anti-NMDAR encephalitis.

## Participants and Methods

### Participants

Fifty (27 males and 23 females) patients with a median age at disease onset of 7.85 years (range: 1.25–14 years) with anti-NMDAR IgG encephalitis were enrolled. All patients were followed up for a duration ranging from 1 to 6 years (2.91 ± 1.21 years). Anti-NMDAR encephalitis was diagnosed based on (1) clinical characteristics–one or more of the following: (a) abnormal mental behavior or cognitive impairment; (b) speech disorder; (c) seizures; (d) involuntary movements; (e) consciousness disturbance; (f) autonomic dysfunction or central hypoventilation; (2) positive cerebrospinal fluid (CSF) anti-NMDAR-IgG antibody, with or without serum NMDAR-IgG antibody positivity; and (3) exclusion of other diseases (febrile seizure, infectious encephalitis, toxic encephalitis, inherited metabolic disease, etc.).

### Methods

We collected information regarding (1) seizure classification (focal seizures, generalized seizures, status epilepticus, or cluster seizures); (2) onset of seizures; (3) consciousness disturbance; and (4) history of herpes simplex virus encephalitis. Lumbar puncture results were reviewed to collect the data regarding CSF white blood cell count and anti-NMDAR-IgG titer. All patients with seizures underwent cranial magnetic resonance imaging (MRI) with T2-FLAIR and prolonged electroencephalograms. The procedure is the same for all patients. MRIs were reviewed to identify the involved brain areas (including the temporal lobe, lateral, frontal, parietal, occipital lobe, hippocampus, cingulate gyrus, brainstem, base section, and thalamus). EEGs were reviewed to identify focal slow-wave, diffuse slow-wave, and interictal epileptiform discharge patterns. First-line treatment consisted of methylprednisolone, human immunoglobulin, and plasma exchange. Second-line treatment consisted of rituximab and cyclophosphamide.

This study was approved by the Capital Institute of Pediatrics Ethics Committee (SHERYXLL). All participants or their legal guardians provided written informed consent prior to enrollment in this study.

### Determination of Autoimmune Antibodies

The serum and CSF examinations of patients were performed by indirect immunofluorescence assays using transfected EU 90 cells (BIOCHIPs, Euroimmun AG, Lübeck, Germany). All specimens were tested for immunoglobulin G antibodies with the following specificities: NMDAR, AMPAR, GABAR, anti-Hu, anti-Ri, and anti-Yo antibodies.

### Definitions of Status Epilepticus and Cluster Seizures

Status epilepticus is defined as a general convulsive seizure lasting more than 5 min, non-convulsive seizure or focal convulsive seizure lasting more than 15 min, or two or more seizures without full recovery of consciousness between them (5–7). Cluster seizures are defined as ≥2 seizures within 24 h. Epilepsy after encephalitis is defined as seizures persisting for 1 year after the diagnosis of encephalitis.

### Statistical Analysis

SPSS (version 22.0; SPSS Inc., Chicago, IL, USA) was used for statistical analysis. Categorical variables were evaluated using the Pearson chi-square or Fisher's exact test. The *t*-test was used for comparisons of continuous variables. The differences were considered statistically significant when *P* < 0.05.

## Results

### Clinical Characteristics of Seizures

Fifty patients were included in the final analysis, of which 34 (68.0%) had seizures. Twenty-two patients (64.7%) had status epilepticus, 20 (58.8%) had cluster seizures, 19 (55.9%) patients had focal seizures, and 15 (44.1%) had generalized seizures. Over at least 1 year of follow-up, the average duration of seizures was 14.6 days (range, 1–47 days), except for three patients with anti-NMDAR encephalitis secondary to herpes simplex virus encephalitis who had persistent seizures.

**Figure 1:**
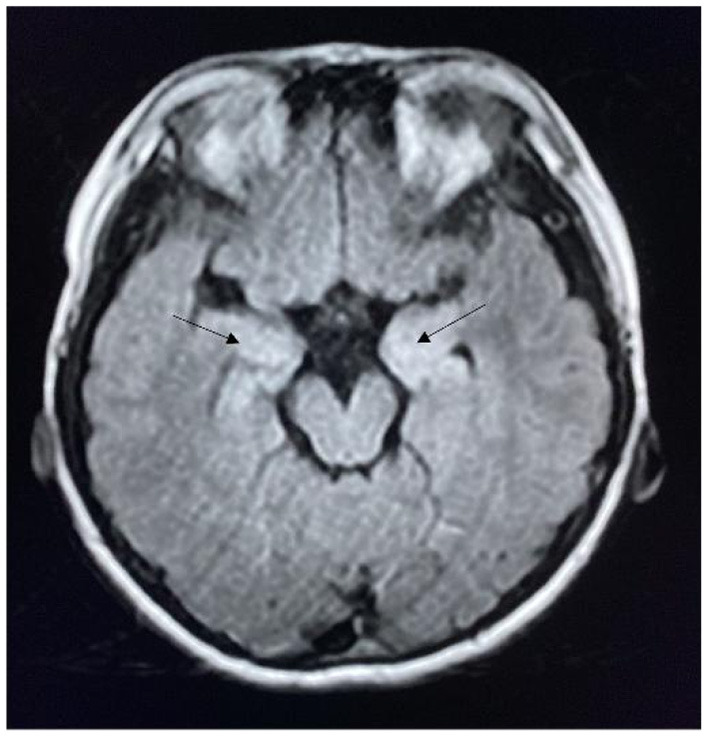
MRI of a patient with anti-NMDAR encephalitis showing bilateral hippocampal lesions (arrow).

**Figure 2:**
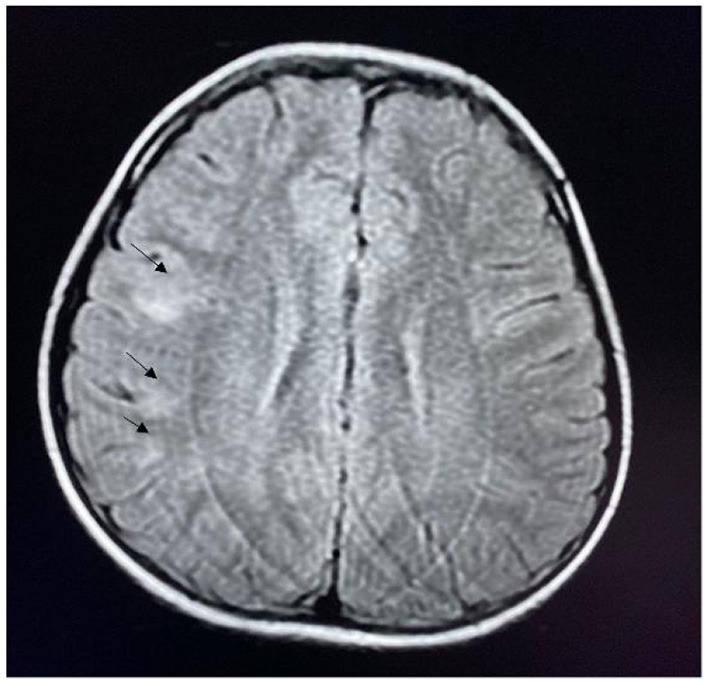
MRI of a patient with anti-NMDAR encephalitis showing lesions in the frontal and parietal cortex (arrow).

The MRI results of the 34 patients with seizures revealed abnormal lesions in 13 (38.2%) patients, including three patients with herpes simplex virus encephalitis with bilateral malacia of the temporal and frontal lobes. In the acute phase, five patients had lesions in the limbic system, and 10 patients had lesions involving the neocortex (Figures 1, 2).

Regarding EEG findings, 27 (79.4%), 23 (67.6%), 6 (17.6%), and 6 (17.6%) patients had bilateral diffuse slow waves (theta wave predominantly), interictal epileptiform discharge, interictal focal slow waves, and extreme delta brush, respectively. Thirteen seizure types were recorded during the EEG monitoring (six focal unaware seizures, three asymmetric tonic seizures, two hypomotor seizures, one epileptic spasm, and two tonic seizures).

Twenty-five (73.5%) children had CSF white blood cells ≥5/mm^3^, and 19 (55.9%) had CSF anti-NMDAR-IgG titer ≥1:100.

All children received first-line treatments such as methylprednisolone, human immunoglobulin, and plasma exchange, and 17 patients (50.0%) received second-line immunotherapy (rituximab or cyclophosphamide). Of the 17 patients who received second-line immunotherapy, six (17.6%) developed anti-NMDAR encephalitis recurrence.

### Comparison of the Clinical Characteristics of Patients With or Without Seizures

The clinical characteristics of patients with seizures (*n* = 34) were compared to those without (*n* = 16). Those with seizures were more likely to have disturbance in consciousness and interictal epileptic discharge on EEG, lower Glasgow coma score, and higher modified Rankin scale (mRS) score (all *P* < 0.05) (Table 1).

**Table 1:** Comparison of clinical characteristics between patients with or without seizures.

	**With seizures**	**Without seizures**	**χ^2^(t)**	***P***
Number and characteristics	34	16		
Gender (female)	18 (52.9%)	5 (31.2%)	2.061	0.151
Glasgow score	11.21 ± 2.29	12.81 ± 1.47	−2.56	0.014
mRS	3.09 ± 1.62	2.0 ± 1.09	2.43	0.019
Consciousness disturbance	20 (58.8%)	3 (18.8%)	7.034	0.008
Psychiatric symptoms	26 (76.5%)	9 (56.3%)	2.118	0.146
Speech dysfunction	6 (17.6%)	4 (25.0%)	0.369	0.544
Movement disorders	23 (67.6%)	11 (68.8%)	0.006	0.938
Cranial MRI with abnormal findings	13 (38.2%)	3 (18.8%)	1.898	0.168
Epileptic form discharge on EEG	23 (67.6%)	4 (25.0%)	9.177	0.002
CSF pleocytosis (>5/mm^3^)	25 (73.5%)	8 (50%)	2.684	0.101
Anti-NMDAR antibody titer ≥1:100	21 (61.8%)	8 (50%)	0.618	0.432
Second-line immunotherapy	17 (50.0%)	4 (25.0%)	2.791	0.095
Underlying teratoma	3 (8.8%)	0	1.502	0.22
After herpes simplex virus type 1 encephalitis	3 (8.8%)	0	1.502	0.22

*EEG, Electroencephalogram; CSF, cerebrospinal fluid; MRI, magnetic resonance imaging; mRS, modified Rankin score*.

### Comparison of Seizures in the Limbic Cortex and Neocortex

Patients were divided into three groups according to the cranial MRI results: non-lesion, limbic system lesion (including hippocampus, amygdala, temporal pole, frontal orbital gyrus, cingulate gyrus, etc.), and neocortical lesion groups. After Bonferroni correction, there was no significant difference between groups (non-lesion group and limbic system lesion group, non-lesion group and neocortical lesion group, limbic system lesion group and neocortical lesion group). Two patients with acute lesions involving the neocortex continued to experience seizures after 1 year. Patients in the limbic system lesion group and neocortical lesion group were more likely to be taking ASMs and receive second-line immunotherapy compared to those in the non-lesion group; however, these differences were not statistically significant (Table 2).

**Table 2:** Analysis of clinical features of seizure between the three groups.

	**No lesion**	**Limbic system lesion involvement**	**Neocortical cortex lesion involvement**	**χ^2^**	***P***
Number and characteristics	34	5	10		
Gender (female)	14 (41.2%)	3 (60%)	3 (30%)	1.25	0.53
Status epilepticus	12 (35.3%)_a_	4 (80%)_a_	7 (70%)_a_	6.18	0.041
Cluster seizures	13 (38.2%)	2 (40%)	5 (50%)	0.8	0.4
Epileptic form discharge on EEG	21 (61.8%)	4 (80.0%)	6 (60.0%)	0.68	0.71
Generalized slowing on EEG	32 (94.1%)	5 (100.0%)	9 (90.0%)	0.59	0.74
ASMs therapy	12 (35.3%)	3 (60.0%)	6 (60.0%)	2.59	0.27
Presence of seizure after 1 year	0	0	2 (20.0%)	–	–
Second-line drugs	11 (32.4%)	4 (80.0%)	6 (60.0%)	5.55	0.06
Relapse	5 (14.7%)	1 (20.0%)	3 (30.0%)	1.21	0.54

*EEG, Electroencephalogram; ASMs, anti-seizure medications*.

*Each subscript letter(a) represents a subset of a group, and there is no significant difference between the groups after Bonferroni correction at the 0.05 level*.

### The Relationship Between Recurrence and Seizures

Compared to patients without seizures, those with seizures had a higher rate of encephalitis recurrence [7 (20.6%) vs. 1 (6.3%)]; however, this difference was not statistically significant (*p* = 0.409) (Table 3).

**Table 3:** Relationship between recurrence and seizures.

	**Number of cases**	**Number of cases with recurrence**	**Number of cases without recurrence**	**Recurrence rate (%)**
With seizure	34	7	27	20.6%
No seizure	16	1	15	6.3%

*p = 0.409*.

## Discussion

Anti-NMDAR encephalitis is one of the most common types of autoimmune encephalitis. The main clinical manifestations are abnormal mental behavior, seizures, memory loss, involuntary movements, and disturbance of consciousness. The underlying mechanism involves binding of the anti-NMDAR antibody to the NR1 receptor, which leads to a decrease in selective and reversible NMDAR density through internalization, and a decrease in NMDAR-mediated synaptic function. Seizures are thought to result from the accumulation of glutamate, which leads to increased excitation of cerebral cortical neurons (6). Compared with adult patients, children with anti-NMDAR encephalitis are more likely to experience seizures (4). In this study, 34 (68.0%) patients had seizures, and focal seizures were the most common (55.9%), which is similar to the previous studies (7–9). Seizures can occur at any stage during the course of the disease and can present as tonic-clonic seizures, complex focal seizures, focal secondary generalized seizures, and status epilepticus. During follow-up, except for three patients with anti-NMDAR encephalitis secondary to herpes simplex virus encephalitis who had persistent seizures, the duration of seizures in the rest of the patients ranged from 1 to 47 days; that is, all seizures occurred within 47 days, which is consistent with the previous reports (9). Therefore, except for epilepsy caused by anti-NMDAR encephalitis secondary to herpes simplex virus encephalitis, long-term use of ASMs is not recommended for other anti-NMDAR encephalitis.

Seizures are the most common presenting symptom of anti-NMDAR encephalitis in children, while adults are most likely to present with psychiatric symptoms (4). Among the 50 patients with anti-NMDAR encephalitis in this study, 34 (68.0%) had seizures, which was consistent with most previous reports (5, 10, 11). A study comparing children with anti-NMDAR encephalitis with or without seizures found that the incidence of disturbance in consciousness and temporal lobe lesions was higher in the seizure group than in the non-seizure group (12), suggesting that the presence of seizures is indicative of a more serious condition. In this study, there were differences in consciousness, Glasgow coma score, mRS, and interictal epilepticform discharge in patients with and without seizures, suggesting that children with seizures were more severely affected.

The use of ASMs alone is less effective for seizures caused by immune encephalitis. Therefore, immunotherapy is the mainstay of treatment for anti-NMDAR encephalitis, and ASMs such as benzodiazepines, sodium valproate, levetiracetam, and lamotrigine are used as a temporary treatment for seizures. Most scholars do not recommend the long-term use of ASMs (13). Of the 34 patients with seizures in this study, 16 (47.1%) did not continue to take ASMs after discharge, compared to 18 (52.9%) who did. Three patients continued to experience seizures 1 year after hospital discharge, and these three patients had anti-NMDAR encephalitis secondary to herpes simplex virus encephalitis. ASMs included oxcarbazepine, levetiracetam, sodium valproate, topiramate, and clonazepam.

There are no reports on the relationship between neocortical lesions and seizures or the relationship between limbic system lesions and seizures in children with anti-NMDAR encephalitis. In this study, compared to children in the non-lesion group, the incidence of status epilepticus was higher among children in the limbic system and neocortical lesion group; this finding suggests that children with cranial MRI abnormalities in these regions are more likely to develop status epilepticus. Moreover, children with lesions in the limbic system and neocortex were more likely to be taking ASMs than those without lesions on cranial MRI. Of note, only patients in the neocortical lesion group still had seizures 1 year after hospital discharge, suggesting that neocortex involvement is more likely to cause epilepsy. Compared to patients with normal cranial MRI findings, those with abnormal findings were more likely to receive second-line immune therapy, indicating that patients with abnormal cranial MRI findings have more severe disease and a slower recovery.

The recurrence rate of anti-NMDAR encephalitis is ~12%, and the risk factors for relapse include an underlying teratoma, serious illness, and delayed immunotherapy (6). Of the 34 patients with seizures in this study, seven (20.6%) had recurrence compared to only one (6.3%) patient in the seizure-free group; however, these differences were not statistically significant, which may be due to the small number of cases.

In summary, children with anti-NMDAR encephalitis are likely to present with seizures. In particular, status epilepticus and cluster seizures are common. The overall prognosis after immunotherapy is good, and most ASMs can be stopped during the recovery period. Children with herpes simplex virus encephalitis secondary to anti-NMDAR encephalitis and neocortical lesions are likely to have refractory epilepsy.

## Data Availability Statement

The raw data supporting the conclusions of this article will be made available by the authors, without undue reservation.

## Author Contributions

JZ contributed in preparing the draft manuscript of this article and prepared the text. JS and PZ prepared the figure. QC was responsible for all supervision. HR was responsible for the detection of specimens. SF and XY have been involved in the management of the children. All authors contributed to the article and approved the submitted version.

## Funding

This work was supported by the Beijing Municipal Administration of Hospital (Grant Number: PX 2019049).

## Conflict of Interest

The authors declare that the research was conducted in the absence of any commercial or financial relationships that could be construed as a potential conflict of interest.

## Publisher's Note

All claims expressed in this article are solely those of the authors and do not necessarily represent those of their affiliated organizations, or those of the publisher, the editors and the reviewers. Any product that may be evaluated in this article, or claim that may be made by its manufacturer, is not guaranteed or endorsed by the publisher.

## References

[1] Dalmau J Tüzün E Wu HY Masjuan J Rossi JE Voloschin A. Paraneoplastic anti-N-methyl-D-aspartate receptor encephalitis associated with ovarian teratoma. Ann Neurol. (2007) 61:25–36. 10.1002/ana.21050 17262855 PMC2430743

[2] Holzer FJ Rossetti AO Heritier-Barras AC Zumsteg D Roebling R Huber R. Antibody-mediated status epilepticus: a retrospective multicenter survey. Eur Neurol. (2012) 68:310–7. 10.1159/000341143 23051892

[3] Gaspard N Foreman BP Alvarez V Cabrera Kang C Probasco JC Jongeling AC. New-onset refractory status epilepticus: etiology, clinical features, and outcome. Neurology. (2015) 85:1604–13. 10.1212/WNL.0000000000001940 26296517 PMC4642147

[4] Titulaer MJ McCracken L Gabilondo I Armangué T Glaser C Iizuka T. Treatment and prognostic factors for long-term outcome in patients with anti-NMDA receptor encephalitis: an observational cohort study. Lancet Neurol. (2013) 12:157–65. 10.1016/S1474-4422(12)70310-1 23290630 PMC3563251

[5] Graus F Titulaer MJ Balu R Benseler S Bien CG Cellucci T. A clinical approach to diagnosis of autoimmune encephalitis. Lancet Neurol. (2016) 15:391–404. 10.1016/S1474-4422(15)00401-9 26906964 PMC5066574

[6] Dalmau J Lancaster E Martinez-Hernandez E Rosenfeld MR Balice-Gordon R. Clinical experience and laboratory investigations in patients with anti-NMDAR encephalitis. Lancet Neurol. (2011) 10:63–74. 10.1016/S1474-4422(10)70253-2 21163445 PMC3158385

[7] Dalmau J Gleichman AJ Hughes EG Rossi JE Peng X Lai M. Anti-NMDA-receptor encephalitis: case series and analysis of the effects of antibodies. Lancet Neurol. (2008) 7:1091–8. 10.1016/S1474-4422(08)70224-2 18851928 PMC2607118

[8] Florance NR Davis RL Lam C Szperka C Zhou L Ahmad S. Anti-N-methyl-D-aspartate receptor (NMDAR) encephalitis in children and adolescents. Ann Neurol. (2009) 66:11–8. 10.1002/ana.21756 19670433 PMC2826225

[9] Wang X Wan J Wei Z Song C Kang X Du F. Seizure outcomes in patients with anti-NMDAR encephalitis: a follow-up study. Epilepsia. (2017) 58:2104–11. 10.1111/epi.13929 29098690

[10] Ho AC Chan SH Chan E Wong SS Fung ST Cherk SW. Anti-N-methyl-D-aspartate receptor encephalitis in children: incidence and experience in Hong Kong. Brain Dev. (2018) 40:473–9. 10.1016/j.braindev.2018.02.005 29599011

[11] Favier M Joubert B Picard G Rogemond V Thomas L Rheims S. Initial clinical presentation of young children with N-methyl-D-aspartate receptor encephalitis. Eur J Paediatr Neurol. (2017) 22:404–11. 10.1016/j.ejpn 29310866

[12] Wu L Hu C Long L Long X Li J Liu W. Clinical features of anti-N-methyl-D-aspartate receptor encephalitis and the concomitant seizure. Zhong Nan Da Xue Xue Bao Yi Xue Ban. (2019) 44:544–8. 10.11817/j.issn.1672-7347.2019.05.011 31303618

[13] de Bruijn MAAM van Sonderen A van Coevorden-Hameete MH Bastiaansen AEM Schreurs MWJ Rouhl RPW. Evaluation of seizure treatment in anti-LGI1, anti-NMDAR, and anti-GABA B R encephalitis. Neurology. (2019) 92:e2185–96. 10.1212/WNL.0000000000007475 30979857 PMC6537134

